# Supplementary material for: Differentiation between mechanically loose and fixed press-fit implants using quantitative acoustics and load self-referencing: A phantom study on shoulder prostheses in polyurethane foam
Source: PLoS One. 2020 May 29;15(5):e0233548. doi: 10.1371/journal.pone.0233548 (PMC7259646; doi:10.1371/journal.pone.0233548)
Supplement: Attachment — Submitted filename: Response to Reviewers.docx [file pone.0233548.s001.docx]

## Differentiation between mechanically loose and fixed press-fit implants using quantitative acoustics and load self-referencing: A phantom study

Florian Vogl^a^, Stefanie Greger^a^, Philippe Favre^b^, William R. Taylor^a^, Paul Thistlethwaite^b^

We would like to thank the Reviewers for their critical feedback to our manuscript, which has helped to improve our manuscript - a brief point-by-point response to Reviewers’ comments is presented below. Wherever possible, we have included the modified text or added text passage for your convenience, with changes highlighted in red.

We have further made our research data and analysis scripts available under https://doi.org/10.3929/ethz-b-000353255.

Reviewer 1
This paper aims to address a clinical problem (measuring implant stability) by using a non-invasive and radiation free technique. Using quantitative acoustics methods, the authors have compared the acoustic characteristics of a loose and fixed implant; showing the possibility of assessing the mechanical properties of the bone-implant interface and identifying between a loose and fixed implant.
A very well written paper. A few minor edits and comments below:

Line 100: "This implant was chosen because achievement of sufficient primary stability is central..." So, does this implant provide high stability?

- We have revised the corresponding section to clarify why we have chosen an uncemented stemless implant for our work. As we are primarily interested in the detection method of loosening and were working with artificial bones, our work does not and cannot provide any results on this specific implant´s performance in-vivo.

We have chosen an uncemented stemless implant [24,25] because in these implants success depends critically on whether primary stability can be achieved (as compared to e.g. cemented implants, where the cement provides additional support). Therefore we considered such an implant as a challenging, but also relevant case in which the presented method might be applied in the future.

Line 113-114, Figure 1 description: "a) labels the engulfing bone cement and b) the access slot for sensor mounting". What is this referring to? Does figure 1 require labelling?

We have removed the second part of the description, which remained from a previous version of the manuscript.

Line 288: "Even though both effects,..." wording is awkward and may be missing a term.

- We have revised the sentence:

While the increase of signal energy transferred from the transducer to a loose implant and the decrease of signal energy transmitted across a loose bone-implant interface independently differentiated loose and fixed implants, the combination of both effects through the use of two sensors on both sides of the interface could be advantageous in more challenging clinical situations.

Discussion: This study has been looking at loose and press-fit fixations. It may be good to comment (in the discussion) on how a cemented fixation would be different to current results.

This is an excellent point. We have added the following section to the discussion:

For the intra-operative assessment of primary stability, the locations of the implant and bone sensors, which have shown the best results, are easily accessible during surgery, and loading for load self-referencing could be applied to the implant by the surgeon. As techniques to measure acoustic waves through the soft-tissues have shown promising results [40,41], our technique may also have the potential to be used for the assessment of secondary stability during post-operative follow-up.

While achievement of primary stability is primary a concern for uncemented implants, such an assessment of secondary stability could potentially be useful for cemented implants as well. However, the presence of cement adds an additional layer of complexity - while only one interface (implant-bone) has to be measured for uncemented implants, for cemented implants the stabilities of two or even three interfaces (implant-cement, cement-bone, potentially implant-bone) need to be evaluated. We believe it likely that the technique could be applied to cemented implants, but further research and development will be needed.
