# Supplementary material for: Differentiation between mechanically loose and fixed press-fit implants using quantitative acoustics and load self-referencing: A phantom study on shoulder prostheses in polyurethane foam
Source: PLoS One. 2020 May 29;15(5):e0233548. doi: 10.1371/journal.pone.0233548 (PMC7259646; doi:10.1371/journal.pone.0233548)
Supplement: Attachment — Submitted filename: Response to Reviewers.docx [file pone.0233548.s002.docx]

## Differentiation between mechanically loose and fixed press-fit implants using quantitative acoustics and load self-referencing: A phantom study on shoulder prostheses in polyurethane foam

## Florian Vogl^a^, Stefanie Greger^a^, Philippe Favre^b^, William R. Taylor^a^, Paul Thistlethwaite^b^

We would like to thank the Reviewers for their critical feedback to our manuscript, which has helped to improve our manuscript - a brief point-by-point response to Reviewers’ comments is presented below. Wherever possible, we have included the modified text or added text passage for your convenience, with changes highlighted in red.

Editor

Your study is really  interesting. However, the title promises to much. Therefore I suggest to add some remarks, e.g. „A phantom study using polyurethane foam mimicking shoulder prosthesis“ or something like that. Furthermore, I recommend to add some Limitation in the abstract as well. From polyurethane foam to clinical application is a long way to go. Nevertheless, you should try it.

We have changed the title following this suggestion, as it makes the title more specific while retaining the context. We have further modified the abstract to make the limitations arising from using a bone substitute material more explicit.

Reviewer 1
The authors have adequately addressed all comments and queries.

We would like to thank the Reviewer for helping us improve our manuscript.

Reviewer 2
This biomechanical study investigates the mechanical stability of an shoulder implant without stem an sawbone model of polyurethane foam. The study shows clearly that a differentiation between loose and stable implants is possible in this biomechanical model by acoustic measurements. The methods and quantitative acoustic measurements are described meticulously.

Loosening of prosthetic replacement is more likely a problem at the lower extremities in hip and knee replacement. Because of this secondary loosening of this replaced joints is related to mechanical load in obese patients (found among others reasons: aseptic/septic loosening).
A shoulder prosthesis in an “unloaded prosthesis”. Early loosening after implantation of a shoulder prosthesis is rare and more related to other reasons: intraoperative fracture/fissure, infection, osteoporosis). The evaluation of sufficient primary stability (called press-fit) of an implantat during the operation is difficult. This is clinical experience of the orthopedic surgeon. Proper preoperative planning of the implants at the template and correct reaming of the bone helps to achieve adequate primary implant stability in the bone.
From clinical prospective good primary stability and press-fit of the implant is important. But various independent factors influence stable osseointegration of the implant. Furthermore, a customized postoperative treatment regimen is necessary avoiding excessive micromotion of the implant in the first week after surgery.

Thank you for these insightful comments – we fully agree that the two primary use-cases we address are somewhat different – depending on the joint and the implant design, either evaluation of primary stability or of secondary stability will have more clinical relevance. While we investigate shoulder implants in this work, we do not want to claim that both use-cases are equally useful for such an implant. While our work cannot directly show that our results generalize to other joints, we think it highly likely. At least for knee implants we have preliminary data showing that the same effects.

We furthermore agree that primary stability is only one requirement for successful osseointegration. Proper planning, skillful execution, and a proper postoperative treatment are necessary as well – and we hope that our manuscript does not give the impression that primary stability is the miracle solution to achieving osseointegration. If you have suggestions where these points need to be clarified, we are more than happy to do so.

As mentioned in the paper evaluation of secondary loosening of implants remains hard to detect. In practice most suitable diagnostic approaches are CT scan and if available SPECT-CT. The examinations should, respectively must, clarify implant loosening before an intervention. This has to be discussed with the patient. CT scan and SPECT-CT are non-invasive.

While both CT and SPECT are certainly diagnostic approaches to detect secondary implant loosening, SPECT requires the injection of a radioisotope and considerable time, and both methods require sophisticated equipment as well as image analysis by experts. As acoustic methods have the potential to address these drawbacks, we believe it worthwhile to research alternative methods. Of course, the uncertainty of the final results is part of the intrigue.

Acoustic techniques require direct contact of piezo-electric actuator and the accelerometer with bone and implant. An intraoperative (invasive) testing is possible. However loose implants are easy to identify by manual testing during the intervention in the operating theatre. The authors should clarify how acoustic techniques can offer simple and non-invasive assessment of prosthetic loosening in approximation to a clinical case to avoid confusion with the biomechanical model and non-invasive techniques.

Acoustic techniques do not require direct contact, but can also be applied to the skin of the patient. Of course, the soft-tissues reduce the signal and introduce additional complexities, but the field of the quantitative bone acoustics has shown that such measurements can be done. Given this, and the fact that acoustic measurements for implant loosening have been successfully performed already, we remain cautiously optimistic.

We have modified the corresponding section in our manuscript to make the potential of this technique clearer:

“As techniques to measure acoustic waves through the soft-tissues have shown promising results [40,41], our technique may also have the potential to be used for the non-invasive assessment of secondary stability during post-operative follow-up in which tranducer and sensor would be placed directly on the skin of the patient.”

A critical point bringing these techniques into clinical practice seems to be the specificity of the acoustic measurements on the implant type, joint und anatomy, which makes interpretation of the measurements difficult. The new approach “load self-referencing (LSR)” with application of a load to the implant changing the acoustic properties of an unstable implant would be a promising further development. This approach would serve as their own reference.

We agree that load-self referencing could be a promising way of circumventing the problem of different implant types, joints or anatomies. Further research will show how far this promise can take us.

But the acoustic characteristics under different loading conditions were not plausible. Further investigations are needed.

We are unsure, whether we have correctly understood this comment: The loading conditions should be the right order of magnitude (as measured in [1]) and the acoustic characteristics under different loading conditions were measured directly.

[1] Westerhoff P, Graichen F, Bender A, Halder A, Beier A, Rohlmann A, et al. In vivo measurement of shoulder joint loads during activities of daily living. J Biomech. 2009;42: 1840–1849. doi:10.1016/j.jbiomech.2009.05.035

Furthermore, translation in clinical practice in the operating theatre seems to be difficult. A loaded versus unloaded position/condition of an extremity (single-legged stance for hip and knee implant?) in the operating theatre is not possible also in respect to aseptic technique and the surrounding soft tissue.

During the operation, the surgeon could apply the load directly to the implant, to which he naturally has direct access. A special instrument could be designed by the implant manufacturer to apply a certain standardized load to the implant - of course, all standard surgical procedures regarding aseptics would have to be respected, but we see no fundamental or particular technical challenge in this respect.
